# Probing of Nucleic Acid Structures, Dynamics, and Interactions With Environment-Sensitive Fluorescent Labels

**DOI:** 10.3389/fchem.2020.00112

**Published:** 2020-02-28

**Authors:** Benoît Y. Michel, Dmytro Dziuba, Rachid Benhida, Alexander P. Demchenko, Alain Burger

**Affiliations:** ^1^Université Côte d'Azur, CNRS, Institut de Chimie de Nice, UMR 7272 – Parc Valrose, Nice, France; ^2^Laboratoire de Bioimagerie et Pathologies, UMR 7021 CNRS, Faculté de Pharmacie, Université de Strasbourg, Illkirch, France; ^3^Mohamed VI Polytechnic University, UM6P, Ben Guerir, Morocco; ^4^Laboratory of Nanobiotechnologies, Palladin Institute of Biochemistry, Kyiv, Ukraine; ^5^Institute of Physical, Technical and Computer Science, Yuriy Fedkovych National University, Chernivtsi, Ukraine

**Keywords:** fluorescence sensing, emissive nucleobase, nucleoside analog, probing nucleic acids, probing interactions

## Abstract

Fluorescence labeling and probing are fundamental techniques for nucleic acid analysis and quantification. However, new fluorescent probes and approaches are urgently needed in order to accurately determine structural and conformational dynamics of DNA and RNA at the level of single nucleobases/base pairs, and to probe the interactions between nucleic acids with proteins. This review describes the means by which to achieve these goals using nucleobase replacement or modification with advanced fluorescent dyes that respond by the changing of their fluorescence parameters to their local environment (altered polarity, hydration, flipping dynamics, and formation/breaking of hydrogen bonds).

## Introduction

Rapid progress in genomics, transcriptomics, and epigenomics are driving a strong demand for reliable fluorescence-based tools for studying the structural and conformational polymorphisms of nucleic acids (NAs), their variability and internal dynamics, their interactions with proteins, metabolites, NA-targeting drugs, water molecules, and ions at sub-molecular and atomic levels (Wilhelmsson and Tor, 2016). Such tools should allow direct molecular recognition between tested NAs and probes that produces an easily recordable and interpretable output signal. They must be viable in different heterogeneous media, including living cells and tissues. Further complicating this task, ideally this technology ought to exclude double labeling of probing NA sequence(s): the requirement for energy transfer, electron transfer quenching or excimer formation between incorporated dyes. The informative signal should be generated by a single fluorescent nucleoside site-selectively incorporated into the probing NA strand and reporting any interactions by varying one of the fluorescence parameters: fluorescence intensity, fluorescence lifetime, optical anisotropy, fluorescence color (emission wavelength) or appearance of new bands in fluorescence emissions (Herdewijn, 2008). Ultra-high sensitivity is insufficient; the fluorescence reporting signal needs to be informative and perceptible to the weak intermolecular interactions that can be expressed as the effects of polarity and H-bonding at the sites of probe location (Demchenko, 2015). In this review, we show that such important developments have already become a reality and we analyze existing and prospective pathways leading to this ambitious goal.

Traditional fluorescence techniques, as well as more recently introduced approaches such as two-photon excitation and single molecular detection, can be useful and efficient here. Since the very weak fluorescence of natural NA bases prevents most applications, the key issue is the design of fluorescence reporters that can be incorporated into a system of interest with minimal perturbation of its structure and dynamics. Hundreds of fluorescent probes are commercially available and are used routinely and more are developed every year. Progress in this area will ultimately depend on the ability of chemists to rationally design fluorophores with parameters that are optimal for the detection methods and, at the same time, possess recognition units for a broader range of applications.

This review focuses on cutting-edge achievements and novel applications for single emissive nucleosides in the study of NA structures, dynamics and interactions. After summarizing both the major problems that can be overcome with this approach and the methodologies for incorporating fluorescent base analogs, we will critically analyze the applicability, achievements and prospects for each of the fluorescence detection methods.

## Resolving Problems With Smart Base Substituents

DNA is the hereditary material in all cellular organisms on Earth. In humans, about 1.5% of DNA encodes for proteins. The remaining 98.5% is non-coding DNA, which is essential for cell functions: non-coding DNA includes the regulatory elements of the protein-coding genes, such as promoters and enhancers, where numerous transcription factors bind to transcriptionally regulate the expression of corresponding genes. Furthermore, a large portion (up to 75%) of the non-coding DNA is transcribed into non-coding RNAs (Djebali et al., 2013). The classical examples are transfer RNAs (tRNAs) and ribosomal RNAs (rRNAs), the central components of the cellular protein biosynthesis machinery. In addition, micro-RNAs (miRNAs) and long non-coding RNAs (lncRNAs) are involved into post-transcriptional regulation of gene expression, shaping and maintaining of the chromatin landscape, and other key biological processes.

The functions of NAs correlate with their dynamics and structures. Although most DNA exists as double-stranded DNA, the single-stranded forms are important intermediates in the processes of DNA replication, transcription, repair, and recombination (Técher et al., 2017). The linear NA chains can also create a variety of tridimensional motifs, which define structural polymorphisms (Kaushik et al., 2016; Strobel et al., 2018). Beyond the well-known B-DNA and A-RNA forms, NA can fold into a Z duplex, triplex, quadruplex, or i-motif, and form bulges, loops, holiday junctions, and pseudoknots, etc. These non-canonical motifs are believed to have an important influence on major cellular processes. For instance, guanine-rich sequences found at the end of chromosomes can fold into G-quadruplexes, which are thought to protect chromosome integrity. RNA exhibits a superior degree of structural complexity and diversity. Many non-coding RNAs contain multiple structural motifs that can interact to build more diverse three-dimensional structures, such as those of tRNAs and ribozymes. NAs are anionic polyelectrolyte macromolecules that must adapt to the crowded cellular environment. Their structures and functions depend on their interplay with the surrounding water and ions, on chemical modifications (e.g., to DNA and RNA bases), pH, temperature and interactions with other NAs (e.g., non-coding RNAs) and/or proteins (e.g., histones and DNA polymerase). NA/NA and NA/protein interactions are central to cellular life and regulation, including replication, repair, recombination, base modifications, transcription, viral infections, and protein synthesis to name a few. For instance, the dynamic interactions between DNA and chromatin proteins and chemical modifications to specific nucleobases (e.g., methylation and demethylation) can dramatically affect gene expression. Above the genetic code, there is a supra code called the epigenetic code, which is involved in cell differentiation, regulation of gene expression, development, and suppression of transposable elements (Bird, 2007; Berger et al., 2009). As one of the major epigenetic modifications, DNA methylation is a multifunctional marker that plays a direct role in the regulation of gene expression and an indirect structural role for the adjacent chromatin, as well as in the expression of miRNAs encoded in the genome (Berney and McGouran, 2018). From its side, miRNA is part of an active RNA-induced silencing complex (RISC) containing multiple associated proteins. It is base paired with complementary mRNA sequences, which leads to mRNA silencing. Other proteins are known to destabilize base-pairing in duplexes and to withdraw a nucleobase from an intra-helical position into a binding pocket. This process corresponds to the so-called base flipping that is common for DNA-repair enzymes and DNA methyltransferases. To understand the functions of NAs, it is therefore important to study the molecular mechanisms, dynamics, conformational changes and interactions of NAs with their cellular partners. However, accessing this information is challenging due to dynamic nature of NAs, the instability of their many structures, and the transitory nature of the associated intermediates.

This overview highlights that methods are urgently needed to survey conformational heterogeneity and dynamics of NAs and for the detection and quantification of NAs and protein/NA interactions. Access to this information is of primary importance for both basic and biomedical research. This may result in new methods for molecular diagnostics, new therapeutic approaches and valuable data for medicinal chemists for the design of drug candidates. To achieve this goal, highly sensitive tools and methods are needed. This is coupled with a move away from traditional radioisotopic methods for NA detection, with a concomitant increase in the use of fluorescence-based technics for identifying NA species. These techniques have become the leading approaches to study complex systems and to visualize living cells due their increased sensitivity, down to single molecule resolution, and strong spatio-temporal detection. They also have numerous applications in high-throughput screening and molecular diagnostics. In this context, fluorescence methods based on single fluorescent nucleosides site-selectively incorporated into NAs have found applications for NA visualization within cells, genotyping, detection of single-nucleotide polymorphisms (SNP), studies of structures, thermodynamics, dynamics of NAs and their interactions with proteins, and small molecules targeting NAs. How do these techniques work, where are we now and what are the prospects for this booming research area?

## Incorporation of Fluorescent Nucleoside Analogs (FNAs): Methodology

Exogenous fluorophores must be introduced into NA structures to perform these analyses (Nakatani and Tor, 2016). These labels need to exhibit high absorption coefficients and quantum yields, and have to be selectively excited in a domain where NAs and proteins are transparent. Fluorophores can be introduced in two distinct ways to make NAs fluoresce: either non-covalent or covalent labeling. Non-covalent labeling uses organic dyes of low molecular weight that bind to NA minor grooves or intercalate helices to increase fluorescence, such as the Hoechst and ethidium bromide dyes, respectively. The vast majority of stains are not sequence specific and many are based on non-covalent attachment to NAs, which are sensitive to conformational changes but do not exhibit specific base recognition (Tatikolov, 2012; Ma et al., 2013). Non-covalently attached fluorophores are mainly used for the visualization of NA in experimental biology procedures (Kapuscinski, 1995; Narayanaswamy et al., 2015). They can also be used to monitor folding and interactions of NAs with their targets but are not able to provide site-specific data and present many other limitations.

**Figure 1:**
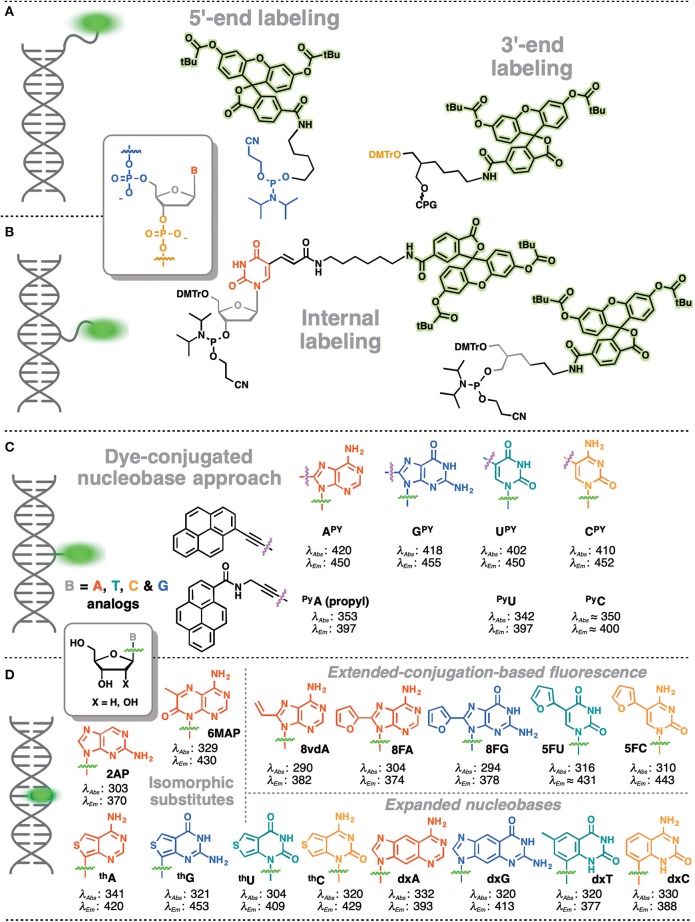
Main strategies for covalent fluorescence labeling of NAs: via a flexible tether either at the 3′/5′-end **(A)**, or at an internal position **(B)** illustrated here by amidite building blocks bearing a fluorescein (Teo and Kool, 2012; Lavis and Raines, 2014); **(C)** through a short and rather rigid linker for extra-helical probing depicted by pyrene, an aromatic polycyclic dye connected to the pyrimidine C5 and purine C8 positions (Rist et al., 2003; Okamoto et al., 2004, 2005a; Saito et al., 2004; Hwang et al., 2005; Seo et al., 2005; Seo and Kim, 2006; Østergaard and Hrdlicka, 2011) and as a nucleobase surrogate for intra-helical probing **(D)** exemplified by selected isomorphic base mimics (Hawkins, 2001; McCoy et al., 2014; Park et al., 2014; Jones and Neely, 2015) and nucleobases made emissive by ring expansion (Gao et al., 2004; Liu et al., 2004; Krueger et al., 2007; Srivatsan et al., 2008a,b; Shin et al., 2011) or extension of conjugation (Gaied, 2005; Greco and Tor, 2005, 2007; Srivatsan and Tor, 2007a,b; Sinkeldam et al., 2008; Greco et al., 2009). Excitation and emission wavelengths were given in nm.

In contrast, covalent labeling can be achieved with high specificity and therefore has a broader range of applications (Davies et al., 2000). The chemical structure of NAs allows for multiple ways of attaching exogenous fluorophores: numerous and diverse examples of this type of labeling have previously been described. The label can be attached to certain position of the NA via a linker or may substitute one nucleobase within the NA (Figure 1). Fluorophores covalently attached to the backbone at one end or within the NA but outside the actual base stack are referred to as external modifications. Substituting a nucleobase inside the base stack is referred to as an internal modification (Wilhelmsson, 2010). External labeling with classical fluorophores, such as rhodamine, cyanine, and fluorescein dyes, linked to the nucleoside via a flexible spacer arm is very common (Figures 1A,B). These dyes are bright and are mainly used for fluorescent detection, for example in DNA sequencing as binary probes when combined with a second partner for Förster Resonance Energy Transfer (FRET). This type of oligodeoxyribonucleotide (ODN) labeling is also used for real time PCR detection, single-nucleotide mutation screening, studying NA conformational changes and NA interactions with target proteins. As an alternative to the FRET-interacting chromophore pairs, the formation or distortion of J-aggregates, H-aggregates, excimers, or exciplexes can be employed to generate changes in fluorescence (Okamoto, 2011; Wilson et al., 2018; and for reviews, see Didenko, 2001; Martí et al., 2007; Varghese and Wagenknecht, 2009; Kolpashchikov, 2010; Guo et al., 2012; Teo and Kool, 2012; Ma et al., 2013).

Despite widespread success, the bi- and multi-fluorophore approaches have several intrinsic disadvantages. Production of DNA or RNA probes bearing two and more different fluorophores leads to synthesis and purification issues that increase the cost by orders of magnitude. For instance, insufficient labeling can produce false results due to the absence of one of the components of the resonance energy transfer pair, which has been the major source of mistakes in FRET-based sensing (Lakowicz, 2006). These issues also apply to other ODN-based probes featuring interactions between two fluorophores. Binary probes display further limitations in structural and functional studies of NAs, for example, they are unable to sense interactions between NAs and their targets if these interactions do not induce sufficient changes in the distance separating the two probes. Further, fluorophores are often bulky and can perturb the system under study. The same comments apply when the binary probe is a NA and a protein.

Fluorescent probes based on emissive nucleoside analogs that are sensitive to the environment bypass double labeling and deserve special attention as powerful tools in bioanalysis, especially given the increasing development of this technology. These DNA-based structures are covalently labeled with a single type of fluorophore and it is possible to attach the probe, not only to a specific NA sequence, but also to position it in the structure in non-perturbing manner. Moreover, it is possible to probe both major and minor grooves in NA double helices. In these efforts, we have observed rapid progress toward technologies designed to address more specific interactions. From the structural point of view, this technology can be divided into categories. The fluorophore can replace one of the natural nucleobases, acting as a nucleobase mimic (Figure 1D). Alternatively, a chromophore (usually a fluorophore) can be grafted with a short linker to one of the nucleobases (Figure 1C) or attached directly to the sugar-phosphate. These fluorescent chemical entities, which are incorporated into the DNA, are usually referred as *fluorescent nucleoside analogs* (FNAs) (Sinkeldam et al., 2010). Depending on their chemical composition, fluorescent nucleobase mimics can be divided into isomorphic, expanded or extended base analogs that maintain or not Wtason-Crick base-pairing, or aromatic fluorophores that lack the H-bonding interactions between complementary bases (Sinkeldam et al., 2010). Due to their well-defined position, FNAs allow site-selective monitoring of conformational or constitutional changes of NAs and are therefore potent signal transducers. FNAs are a group of chemically diverse compounds that often (but not always) share partial deoxyribose moieties with natural nucleosides. A few hundred different FNAs have been described to date. This review is not intended to be a comprehensive catalog of all existing literature reports on FNAs: previous reviews are available on the topic (Asseline, 2006; Wilson and Kool, 2006; Dodd and Hudson, 2009; Srivatsan and Sawant, 2011) and the following comprehensive reviews are especially recommended (Sinkeldam et al., 2010; Wilhelmsson, 2010; Xu et al., 2017; Saito and Hudson, 2018).

The use of FNAs requires the site-selective location of the label within NA. Such a labeling has been well-described and is a methodologically mature area in bioorganic chemistry. Labeling can be accomplished according to different strategies. FNAs can be introduced using either solid-phase phosphoramidite chemical synthesis (Hogrefe et al., 2013) or enzymatic (polymerase) incorporation (Hocek, 2019). FNAs can be formulated as building blocks for the construction of NAs or can be introduced post-synthetically using bioorthogonal chemistry (post-synthetic labeling; Xu et al., 2017).

## Operation With Parameters of Fluorescence Emission

Fluorescence is the emission of light that can occur when a molecule absorbs a light quantum to become excited to the singlet state and then releases its energy and relaxes back to the ground state. Its essential features are the delay in time between light absorption and emission resulting in fluorescence decays in the picosecond (ps) to nanosecond (ns) time range and also the decrease in energy of emitted quanta (the Stokes shift). The time delay allows one to observe different processes occurring on this very short time scale, such as translational or rotational diffusion or resonance energy transfer. The Stokes shift enables not only the separation of the fluorescence signal from that of light scattering in detection devices but can also be the source of valuable information on the intermolecular interactions decreasing the excited-state energy.

The output signal is always the number of emitted light quanta that can be integrated and presented as fluorescence intensity. When the intensity is recorded as a function of time, its decay can be characterized by the fluorescence lifetime and gives rise to lifetime-based sensing. Excitation with linearly polarized light also results in polarized emission (optical anisotropy). Any rotational motion or transfer of energy to another fluorophore decreases the polarization. When the light intensity is recorded with spectral resolution, the resulting fluorescence emission spectra carry the information on the fluorophore interactions and reactivity in the excited state (that may lead to the generation of new bands), which, in turn, opens a new channel of information in sensing.

**Figure 2:**
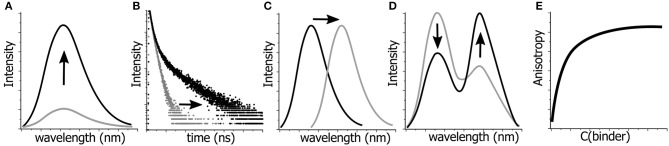
Possibilities for monitoring the changes in molecular structures, dynamics and interactions by fluorescence spectroscopy. Variations in: **(A)** fluorescence intensity, **(B)** fluorescence lifetimes, **(C)** position of the emission maximum, i.e., emission color, **(D)** ratio of intensities at two wavelengths, and **(E)** fluorescence anisotropy can be detected and quantified.

Thus, fluorescence offers a very limited number of parameters for quantitative detection of changes in a given system in terms of their structure, dynamics, and interactions (Figure 2). These include fluorescence intensity at a certain wavelength, fluorescence lifetime, fluorescence anisotropy, position of the emission maxima and, in special cases, the ratio of intensities at two selected wavelengths (Demchenko, 2010). All these parameters can be used in the creation of NA-based probes (Su et al., 2012). In the following sections, we discuss the use of FNAs as the fluorescent reporters that change these parameters in different contexts involving NAs. For each of them, we focus on structural requirements and sensing mechanisms that influence the fluorescence response. The outlined principles can be useful for the rational design of innovative NA-based fluorescent molecular probes.

The choice of detection method should be dictated by the aim of the research project or by the new technology to be developed. For instance, the double-helical conformation can be detected in a simple way, by increasing the intensity of intercalating dyes. Regarding intermolecular interactions and the conformational changes that accompany them, the appropriate choice of sensing method should be selected from those listed above, with an optimal design of the fluorophore and its proper incorporation into the desired sites. Thus, a hybridization assay should generate a strongly distinguishable signal upon binding or non-binding of the target DNA sequence. Ideally, this signal would be an OFF-ON switch in fluorescence intensity. For more complicated issues, such as the atomic-scale variations in structure (hydration), more sophisticated tools are required.

## FNAs for Intensity-Based Sensing

Fluorescence intensity is by far the most widely used parameter of fluorescence emission. It is a relative numerical representation of the number of emitted photons recorded by the detector. Since this parameter depends on fluorophore concentration and many instrumental factors (such as light source brightness, detector sensitivity and illuminated volume), its quantitative determination requires a reference. Within a single experiment series, all parameters related to the instrumental settings are typically kept constant and thus, all changes in fluorescence emission can be attributed to the changes in quantum yield (Φ). The latter is the absolute measure of fluorescence ability at given conditions that can be defined as the ratio of emitted quanta to the whole number of absorbed quanta. Accordingly, since it depends on the relative ratio of efficiencies of emissive and non-emissive excited state depopulation pathways, Φ can vary in broad ranges and is affected by numerous factors, such as the presence of molecular segments with rotational mobility, the formation or breaking of hydrogen bonds and the presence of internal or external quenchers. Hence, fluorescence intensity could change dramatically. Only in the ideal case of complete absence of non-radiative processes, Φ value will reach 100 % and the intensity will become maximal. Usually, with NA base substitutes, Φ is revealed to be much lower. In this respect, if complete quenching (Φ ≈ 0) is achieved, this may determine the broad dynamic range of an assay based on “OFF-ON” switching. The fluorescence intensity changes are usually measured at a single wavelength (usually the band maximum), which is the simplest and most convenient method to get information from a fluorescence reporter.

Many known FNAs have been shown to change their emission intensity in response to different molecular events such as stacking with the flanking nucleobases, forming hydrogen bonds with a complementary nucleobase, etc. Deep knowledge and understanding of the nature of different quenching and lightening mechanisms are beneficial for the rational design of new fluorescent probes.

### FNAs Quenching Upon Stacking

Purines and pyrimidines are known to quench fluorescence of many organic fluorophores. A classic example is the quenching of tryptophan fluorescence by nucleobases within DNA–protein complexes, which is the basis of the first method used to observe DNA–protein interactions by fluorescence spectroscopy (Lakowicz, 2006). In view of this, it is not surprising that the fluorescence of many FNAs are quenched upon their incorporation into DNA. This effect has been widely used in the construction of conformation-sensitive fluorescent probes. FNAs that are quenched upon incorporation into DNA or RNA segments are a family of structurally diverse compounds. Selected examples will be discussed below. Of all FNAs, quenching of 2-aminopurine (**2AP**) has been the most widely studied because it has been the most popular probe for decades (Figure 1D). **2AP** can form stable hydrogen-bonded base pairs with T and C, thus, it can be used as an A and G mimic (Sowers et al., 1986; Law et al., 1996; Millar, 1996; Reha-Krantz et al., 2011). Incorporation of **2AP** introduces minimal, although notable perturbations to the dynamic and thermodynamic stability of double-stranded DNA (Dallmann et al., 2010). The absorption of **2AP** overlaps the red edge of the NA absorption band and its emission shows a relatively large Stokes shift (Figure 1D). In a free form, **2AP** is brightly fluorescent in water (Φ = 0.68). Meanwhile, upon incorporation into ODNs, the fluorescence of **2AP** is severely quenched, reaching up to a 200-fold reduction (Ward et al., 1969).

**2AP** is quenched by all four natural bases with G being the most efficient quencher (Somsen et al., 2005). Similarly, FNAs can be quenched by all four canonical DNA nucleobases or selectively by a few of them. The degree of quenching typically depends on the DNA conformation and the sequence context. Many fluorophores are quenched by Photoinduced Electron Transfer (PET) mechanisms. Upon PET quenching, FNAs can be either reduced or oxidized to form a charge-transfer (CT) complex with one of the surrounding nucleobases and further undergoing radiationless relaxation to the ground state. The PET efficiency is given by the Rehm–Weller equation (Rehm and Weller, 1970; Farid et al., 2011), and the possibility of PET quenching can be estimated by a comparison of the redox potentials of the natural nucleobases with that of the corresponding FNA (Seidel et al., 1996; Psciuk et al., 2012). Among the different canonical nucleobases, guanine, with the lowest oxidation potential, is known to be the most efficient electron donor in PET resulting in strong quenching of many organic dyes upon contact with the G base (Seidel et al., 1996; Torimura et al., 2001). Other mechanisms have been proposed to explain FNA quenching, such as the formation of dark non-emissive states via mixing and delocalizing of molecular orbitals of the fluorophore among neighboring nucleobases, as it has been proposed for **2AP** and 8-vinyladenosine (**8vdA**, Figure 1D; Gaied, 2005). In general, the quenching mechanisms are complex and may involve multiple non-radiative relaxation pathways. The relative contribution of these quenching mechanisms in DNA is not fully understood, even for **2AP**. Despite these gray zones, many FNAs of this group are effectively used in fluorescence sensing.

FNAs that are strongly quenched upon incorporation into ss- and ds-NAs can be used for the development of fluorogenic probes. Within this approach, the “OFF” state is represented by a DNA construct incorporating the emissive nucleoside analog quenched by the neighboring bases. Transition of the fluorescence signal results in the “ON” state, which is observed upon binding of the target (NA, protein or small molecule). A representative set of examples of such probes is given below.

#### Probing the Single-Nucleotide Polymorphism

**Figure 3:**
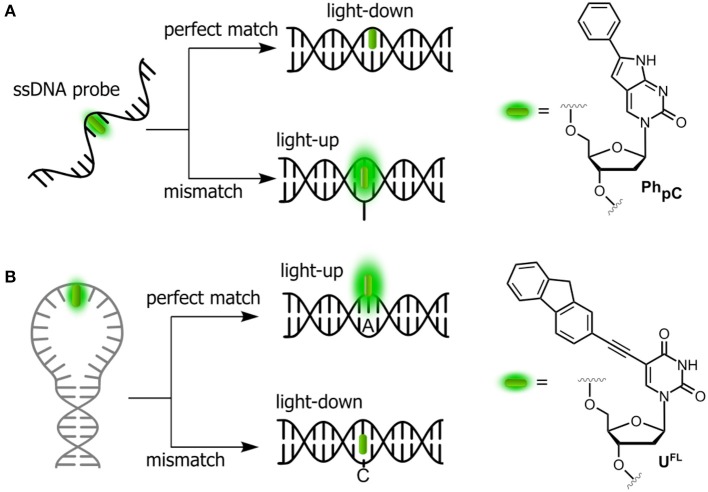
Single-dye hybridization probes for the detection of SNPs. **(A)** Pyrrolocytosine nucleobase allowing mismatch discrimination by a fluorescent turn-on response; and **(B)** quencher-free molecular beacon approach revealing match cases by a light-up signal.

SNPs, also known as single-base mutations, are the most prevalent genetic variations (White and Cantsilieris, 2017). Oligonucleotides incorporating stacking-sensitive nucleosides have been studied for direct probing of single-nucleotide polymorphisms (SNPs), and pyrrolo-cytosines (**pC**, C mimic) are representative for this approach. Typically, the fluorophore is positioned in the probe strand at the position opposite to the SNP site. Hybridization to a perfectly matched target causes the fluorophore to stack between the surrounding nucleobases. A light-down response (OFF) is observed in this case (Figure 3A). However, in the case of mismatch, a perfectly stacked conformation cannot be achieved and a light-up response (ON) is observed (Hudson and Ghorbani-Choghamarani, 2007). Numerous SNP-detecting probes showing such mismatch detection responses have been described (Okamoto et al., 2005a; Dodd and Hudson, 2009), including the aromatic anthracene probe (Duprey et al., 2011). Notably, anthracene is sensitive enough to report single point variants (Zhao Z.-Y. et al., 2012; Duprey et al., 2018) as well as to discriminate cytosine from 5-methylcytosine and even the challenging 5-hydroxymethylcytosine (C vs. 5 mC and 5 hmC; Duprey et al., 2011, 2016). The quencher-free molecular beacon developed by Kim et al. proposes the reverse strategy to distinguish a fully complementary strand from a mismatched target (Hwang et al., 2004; Ryu et al., 2007). For this purpose, a deoxyuridine derivative connected to fluorene at position 5 (**U**^**FL**^, Figure 3B) was used as fluorescent probe and incorporated into the loop region of a hairpin. The beacon signal exhibited a 2-fold increase and a 6-fold decrease upon hybridization with fully complementary and single-base mismatched ODNs, respectively.

#### Detection of Protein-NA Interactions

**Figure 4:**
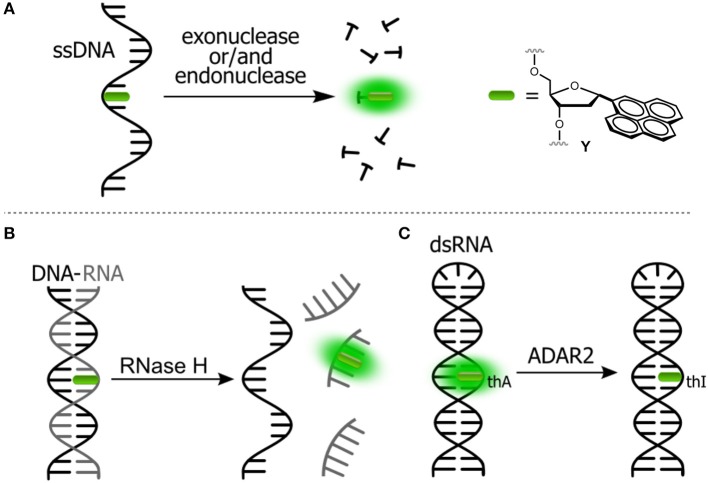
Fluorogenic probes for sensing interactions with enzymes based on NAs labeled with a single FNA: **(A)** ssDNA probe for endo- and exonucleases; **(B)** ds-RNA/DNA probe for RNase H; and **(C)** hairpin RNA probe for adenosine deaminase.

Assays based on FNAs can be used for real-time sensitive detection of protein–DNA interactions including monitoring the activity of different enzymes (Dai and Kool, 2011). A number of enzymes are known to break down the integrity of NAs via hydrolysis of the phosphate diester links. For instance, nucleases are responsible for the degradation of undesirable DNA and RNA fragments. A viral integrase cleaves short oligonucleotide fragments from the 3′-end of double-stranded DNA to prepare for the integration of the DNA into the genome of host cell. The activity of enzymes can be monitored by DNA and RNA-based probes incorporating emissive nucleoside analogs. Representative examples are shown in Figure 4.

The intact form of the probe incorporates one of the nucleosides sensitive to stacking interactions. The enzyme hydrolyzes the NA strand, thus uncaging the fluorophore. Kool et al. developed a set of nuclease probes basing on the pyrene nucleoside (**Y**, Figure 4A). They exploited the fact that pyrene can be quenched by a neighboring thymine, but not quenched by a flanking adenine. By constructing different combinations of pyrene, T and A on a DNA scaffold, they obtained a set of fluorogenic chemosensors for distinct classes of nucleases (Jung et al., 2013). Oligonucleotides 5′-AYT, 5′-YT(A)_8_ and YTTY were designed to be the probes for 3′-exonucleases, 5′-exonucleases and endonucleases, respectively. The oligonucleotides were tested using RNase T, RecJ_f_ and nuclease S1 as representatives of different classes of nucleases. All nucleotides exhibited a significant light-up response, ranging from 40- to 250-fold, upon interaction with their targets (Figure 4A). The rise of fluorescence is caused by the cleavage of Y-T pairs. Remarkably, all nuclease probes were found to be highly selective for the assigned targets, allowing selective detection of nucleases. This assay was applied to measure the nuclease activities in biological liquids (Jung et al., 2013). Hudson et al. described an RNase assay (Figure 4B) based on 6-phenylpyrrolocytidine (^**Ph**^**pC**, Figure 3A). The modified riboside was incorporated into RNA–DNA hybrids in which its emission was quenched. The cleavage of the RNA strand was observed in real time as the fluorescence intensity increased (Figure 4B). The method was reported to be superior to molecular beacon-based detection in terms of cost and sensitivity (Wahba et al., 2010).

**Figure 5:**
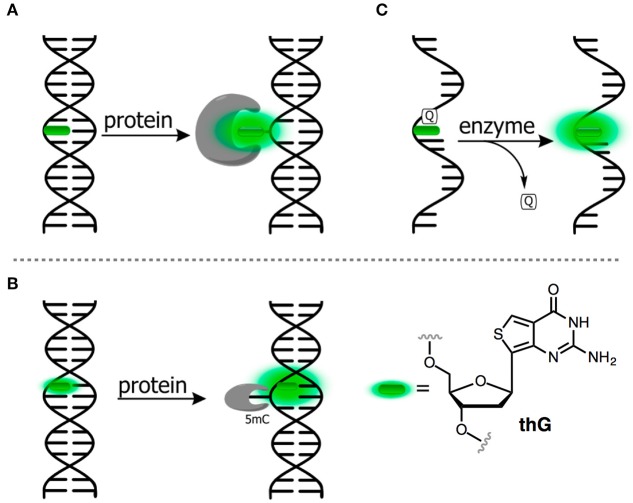
FNAs as a base mimic and stacking-sensitive reporter **(A,B)** for sensing single-base flipping; **(C)** for sensing DNA damage and repair. Q, quencher being either U or 1-methyladenine.

Understanding, at the molecular level, the dynamics and functions of enzymes in interactions with their DNA targets is of primary importance in biology and medicine. Enzymes that catalyze reactions at specific sites in DNA, primarily face the substantial problems of finding the specific site and then forming specific interactions to obtain the catalytically productive enzyme-substrate complex (Stivers et al., 1999). FNAs become the instruments of choice for real-time monitoring of fluctuations and distortions to normal DNA structure that occur in many cases, over a short timescale (Figure 5).

Allan and Reich reported one of the first **2AP**-based assays for monitoring the single-base flipping caused by interaction with the enzyme under steady-state conditions. To do so, one of the adenines within the recognition site of EcoRI DNA methyltransferase was replaced by a **2AP** residue (Figure 5A). A 14-fold increase in fluorescence intensity with a 10-nm blue shift was observed upon interaction with the protein, indicating flipping of **2AP** into the hydrophobic binding site (Allan and Reich, 1996). A more detailed picture of enzymatic mechanisms can be attained when the reaction is investigated using stopped-flow techniques and under pre-steady state conditions (Fedorova et al., 2010). Tor et al. have introduced derivatives of thieno nucleobases with the aim to obtain mimics as close as possible to the structure of all four of the natural bases (Figure 1D). Among these was thienoguanine (**thG**, Figure 5B), which, when incorporated into DNA, can faithfully substitute a G residue while remaining emissive, in contrast to **2AP** (Shin et al., 2011; Sholokh et al., 2015a, 2016). **thG** was incorporated in hemi-methylated DNA and was used to report the flipping of a neighboring methylcytosine into the binding pocket of ubiquitin-like containing PHD and RING finger domains 1 (UHRF1), a key protein involved in the replication and maintenance of the DNA methylation pattern (Kilin et al., 2017). Notably, **2AP** failed to monitor such base flipping. The adenosine analog (**thA**, Figure 1) was introduced in RNA strands and employed to analyze Adenosine Deaminase Acting on RNAs 2 (ADAR2, Figure 4C). This enzyme catalyzes deamination of adenosine into inosine with selectivity for RNA duplexes containing A. Inosine can substitute guanosine, often leading to codon changes in mRNA. The fluorescent **thA** was processed by ADAR2 as the natural nucleobase to give the corresponding analog of the inosine product. The product showed a reduced quantum yield, therefore enabling the monitoring of the reaction progress and facilitating mechanistic studies of RNA editing (Mizrahi et al., 2015).

#### Unraveling the Mechanisms of DNA Reactions With Chemicals, Photodamage, and Repair

DNA nucleobases can accumulate chemical damage upon exposure to exogenous or endogenous factors. The most important exogenous factors are genotoxic chemicals and UV-irradiation. Endogenous factors include reactive oxygen species (ROS) and other reactive chemicals, among others (De Bont and van Larebeke, 2004). Furthermore, the intrinsic DNA instability leads to the spontaneous formation of lesions under near-physiological conditions. Examples of DNA lesions include the formation of thymine photodimers, hydrolytic depurination leading to the abasic site lesions, oxidation of guanine to give 8-oxoguanine, hydrolytic deamination of cytosine and 5-methylcytosine leading to C → U, 5mC → T mutations and alkylation of G, to name a few (Schärer, 2003; Gillet and Schärer, 2006). Assays that are capable of detecting and quantifying DNA damage are important for the study of diseases caused by DNA damage, such as cancers. FNAs quenched within the DNA structure are perfect tools for site-selective detection of DNA damage and repair due to their high sensitivity to the local electronic structure of neighboring nucleobases.

Kool et al. reported a fluorescence assay for uracil-DNA glycosylase (UDG), an enzyme that participates in DNA repair by removing uracil from DNA (Ono et al., 2012). A fluorogenic NA substrate was constructed by placing the pyrene **Y** as a nucleobase substitute between two uracil residues within the NA-based probe such that the pyrene emission is strongly quenched by the flanking pyrimidine residues (Figure 5C). The activity of UDG afforded a cleavage of the uracil from the DNA strand. This cleavage was observed by the increased pyrene fluorescence. In another example, the ability of the positively charged 1-methyladenine to quench a pyrene nucleobase was used to design probes to monitor DNA repair (Figure 5C). The intracellular activity of the demethylation enzyme, ALKBH3, which is involved in tumor genesis, was measured using this probe (Beharry et al., 2016; Xu et al., 2017).

#### FNAs in Signaling Aptamers

The design of NAs with specific target binding properties (aptamers) has greatly broadened the application of NA fluorescent probes to the detection of a large variety of analytes, including small molecules, proteins, ions, and even whole cells (Juskowiak, 2010; Gustmann et al., 2018). The recognition properties of aptamers and the sensing properties of emissive nucleoside analogs were fused at the beginning of the 2000s to form a new strategy in the design of fluorescent biosensors (Jhaveri S. D. et al., 2000; Jhaveri S. et al., 2000). FNAs that are quenched by stacking interactions with nucleobases are especially useful in this respect. They can be used in a single-dye format, which simplifies the production of such aptamers. Due to their strong sensitivity to the conformation of NA strand, even minor conformational changes occurring upon binding can be detected. Katilius et al. explored the capability of FNAs to generate binding-specific fluorescence response (Katilius et al., 2006), and found that binding to the target can induce an increase in the fluorescence signal of up to 30-fold in the case of a thrombin aptamer incorporating the pteridine, **6MAP** (Figure 1). The approach was suggested to be of general use for the development of signaling aptamers targeting other proteins. Li et al. described an aptamer-based fluorescent probe featuring competitive hybridization with a **pC**-containing strand. The intact form of the probe was constituted by a double-stranded NA containing a sensing aptamer strand and a **pC**-containing signaling strand, where **pC** fluorescence was strictly quenched. The binding to the target released the signaling strand, providing a light-up fluorescence response (Li et al., 2010).

### FNAs With Segmental Mobility

**Figure 6:**
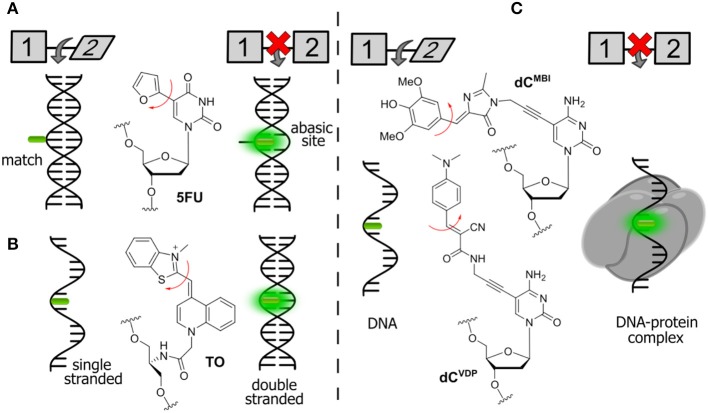
Selected examples of FNAs featuring segmental mobility and their applications in sensing. **(A)** Furan-decorated uridine used as an abasic site sensor; **(B)** peptide-type FNA becoming emissive by forced intercalation; and **(C)** bearing flexible dyes, cytidine FNAs affording fluorescence enhancement upon protein interactions.

The group of fluorophores known as fluorescent molecular rotors, are composed of two or more aromatic rings connected by a few flexible chemical bond, exhibit low quantum yields in solutions at room temperature because of the free internal rotation that serves as a radiationless relaxation channel. In a rigid environment, increased quantum yields are observed due to the restricted intramolecular rotation. Several FNAs are known to exhibit low fluorescence in the “OFF” state due to internal rotation and show an increase in the fluorescence intensity after packing or increased viscosity leading to an “ON” state (Figure 6). They have been used for the detection of abasic sites in DNA, for the construction of hybridization probes and for the sensing of both protein–DNA interactions and local viscosity.

Tor et al. described a series of emissive pyrimidine analogs containing a furan ring linked to their position 5 (Figure 6A). Hybridization of a single-stranded DNA incorporating a furan-modified deoxyuridine (**5FU**) increased fluorescence upon hybridization to a complementary DNA strand containing an abasic site complementary to the fluorophore (Greco and Tor, 2005). Hybridization to the fully complementary strand (with no abasic site) resulted in a decrease of fluorescence. These results were rationalized using a molecular rotor model (Sinkeldam et al., 2011). Rotation around the single bond between uracil and furan was proposed as the principal radiationless relaxation pathway. When the nucleoside faces an abasic site, it becomes sandwiched between the two neighboring Watson-Crick base pairs, which restricts its conformational freedom and therefore increases the fluorescence. When the nucleoside faces an adenosine on the fully complementary strand, the furan becomes exposed to the major groove, where its rotation is no longer restricted.

The Seitz group reported the construction of forced intercalation probes (FIT probes), which were originally designed as single-stranded PNA (Peptide Nucleic Acid) strands containing one cyanine dye (such as thiazole orange, **TO**) as a nucleobase surrogate (Figure 6B). FIT probes were smartly utilized as light-up fluorescent hybridization probes for sensitive detection of DNA and RNA (Köhler et al., 2005). In a single-stranded form, the probes incorporating **TO** exhibit low fluorescence. The formation of the probe–target duplex results in a well-ordered structure, where the fluorophore is forced to intercalate between flat aromatic nucleobases. The FIT probes were found to be useful for the detection of DNA in quantitative Polymerase Chain Reaction (qPCR) and for wash-free detection of RNA in cells and tissues (Socher and Seitz, 2008; Kummer et al., 2011; Hövelmann et al., 2012, 2013).

A similar approach can be used for the detection of protein–DNA interactions. Binding of proteins is known to increase the quantum yield of cyanine dyes attached to DNA due to an increase in local viscosity. This phenomenon is known as protein-induced fluorescence enhancement (PIFE; Hwang and Myong, 2014). It was further utilized by the Hocek group with a series of new FNAs suitable for the detection of protein–DNA interactions. In the first example, a GFP-like fluorophore (**dC**^**MBI**^) was incorporated into DNA (Figure 6C). Fluorescence titration experiments showed that the fluorescence of DNA conjugates increased more than 2-fold upon addition of the single-strand binding protein from *E. coli* (SSB) and transcription factor p53 (Riedl et al., 2012a). In the second study, an improved fluorogenic dCTP analog (**dC**^**VDP**^) was reported, in which the GFP-like fluorophore was substituted by cyanoacetamide-based fluorescent molecular rotors (Figure 6C). Single-stranded DNA labeled with this fluorophore exhibited around 4-fold fluorescence enhancement upon binding to SSB (Dziuba et al., 2015).

### Merits and Limitations of Intensity-Based Sensing

As we have described above, the options for fluorophores that satisfy the requirements of intensity sensing are rather broad. The advantages of this method include the overall simplicity of fluorescence measurements and the adaptability of the instrumentation for developing integrated detection devices and micro-arrays. Due to high sensitivity, and since all the emitted light quanta can be collected over the spectrum with any polarization or time delay, this method is very popular. However, intensity-based sensing has intrinsic drawbacks. Fluorescence intensity is always represented in arbitrary units and the results are not precisely reproducible if measured on different instruments. Furthermore, it is strongly dependent on the concentration of the fluorescent molecular probe in the test system, which is often not exactly known. This raises the problem *of calibrating the sensor element*, which may be not so simple. In addition, the effects of light absorption, light scattering, photobleaching and/or time-dependent degradation of the sensor affinity can be difficult if not impossible to compensate or to calibrate. These difficulties have therefore stimulated intense efforts from researchers in proposing new dyes, detection, and imaging methods intended to correct or exclude these limiting factors. In contrast, sensing methods based on lifetime, anisotropy, and ratiometric emission have strong potential because they give analytical signals that are independent from instrumental settings and probe concentrations (Gryczynski et al., 2003; Demchenko, 2005b, 2014).

## FNAs for Lifetime-Based Sensing

After the initial electronic excitation of molecules, fluorescence develops in the sub-nanosecond to nanosecond time range. The decay function of the fluorescent light emitted by these molecules is essentially concentration-independent and, therefore, no reference is needed. For independent and structurally identical fluorescent molecules, the decay time of excited states typically follow a single exponential function of time, like the decay of radioactive compounds. However, in the case of fluorophore interactions, dynamics, and various reactions, the emission rates can become heterogeneous. As a consequence, the complex multiexponential fluorescence decay profiles of fluorophores incorporated into DNA are frequently observed. NAs can adopt multiple conformations simultaneously and can exhibit a specific combination of different lifetime components corresponding to different subpopulations. Therefore, the lifetime can become the source of valuable information. Resolving the ultrafast decay profiles of **2AP**-labeled DNA and RNA provides information about the site-specific dynamics and heterogeneity of the existing conformations of NAs including those with abasic sites and mismatched base pairs, as well as triplexes (Nordlund et al., 1989; Guest et al., 1991; Edward et al., 2001; Ramreddy et al., 2007, 2009; Xia, 2008; Zhao and Xia, 2009).

**2AP** has been widely used in lifetime-based NA research. It exhibits mono-exponential decay in water, whereas, upon incorporation into DNA, the decay is resolved as a sum of four exponential components with typical lifetimes ranging from 10 ps to 10 ns (Neely, 2005). These lifetime components are thought to reflect different degrees of stacking and conformations. The shortest lifetime component is the most significant and is typically a strongly quenched **2AP**-stacked conformation. The other species represent small populations of partially stacked and unstacked **2AP**. The longest lifetime component was assigned to unstacked **2AP**, which is highly emissive, with a conformation, in which **2AP** protruded from the contact with the natural nucleobases. These minor species are readily detected because their fluorescence is much higher than that of stacked base pairs. In addition to distinguishing different conformations, time-resolved fluorescence can also be used to analyze the variation of their relative populations. Thus, **2AP** can serve to probe site-specific transitions of DNA conformations. For instance, the measurements of fluorescence lifetimes and steady-state experiments of **2AP** were used to register the transitions in a DNA duplex at temperatures significantly below the transition temperature for melting (T_m_; Xu et al., 1994). A dynamic pre-melting transition involving increased exposure of **2AP** to water was observed at temperatures more than 10°C below T_m_.

**Figure 7:**
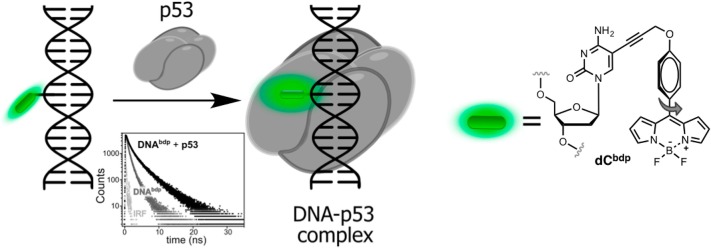
Sequence-specific binding of the transcription factor p53 to DNA sensed via lifetime experiments. Fluorescence decays of labeled DNA before (light gray) and after (dark gray) binding to p53.

**2AP** was also employed as a lifetime probe for studying the kinetics and thermodynamics of conformational changes of DNA or RNA upon binding to proteins (Nguyen et al., 2011). In particular, a time-resolved technique was used to monitor flipping of **2AP** within complexes of double-stranded DNA with the DNA methyltransferases M.HhaI and M.TaqI. In the double helix, the short-lifetime component of the stacked **2AP** was determined to be the major contribution in the decay profile. Upon binding to the enzyme, the disappearance of the short-lifetime component, together with an increase of the long lifetime component was used as an indication of flipping of the fluorescent adenine mimic into the protein-binding site. The interpretation of time-resolved fluorescence decays was supported by X-ray crystal structure analysis (Neely, 2005; Lenz et al., 2007). However, **2AP** is excited with short-wavelength UV light (300 nm) and exhibits very low brightness in DNA, which is incompatible with live-cell imaging. Recently, a BODIPY moiety connected to dC absorbing and emitting light in the blue-green region of the visible spectrum was developed as a molecular rotor (Figure 7). When introduced to DNA, the probe responded upon binding to transcription factor p53 by increasing of fluorescence intensity and lifetime (from 0.8 to 2.1 ns). The latter properties were exploited to visualize the local viscosity using fluorescence-lifetime imaging microscopy in living cells (Dziuba et al., 2016a).

## FNAs for Anisotropy-Based Sensing

**Figure 8:**
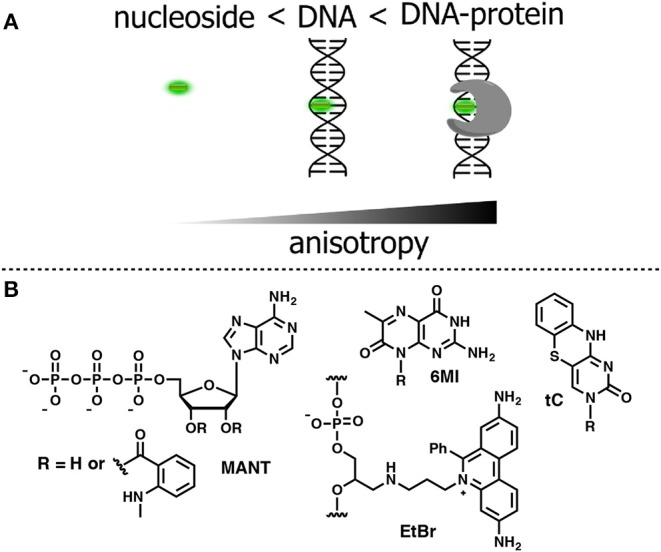
**(A)** Relative changes in anisotropy for increasing molecular sizes of the dye-containing complex. **(B)** FNA suitable for anisotropy-based sensing.

Anisotropy describes the extent of polarized emissions. It can be created by using polarized light that excites only those fluorophores with definite directions. The preferentially excited fluorophores are those that have their absorption transition moments align with the electric field vector of the incident polarized light. After excitation, the emission can become polarized if the fluorophores are immobile on a time scale of fluorescence decay (*high anisotropy*). Rotational diffusion may randomly change the orientation of the fluorophore transition moment, producing emitted light in all space directions and causing depolarization (*low anisotropy*). However, if the size of the rotating unit or the local viscosity increases during the operating experiment, then the rotation will decrease resulting in increase of fluorescence anisotropy (or polarization). The fluorescence lifetime τ_*F*_ is an important parameter that defines the time window for rotation. A larger τ_*F*_ will allow more time to rotate and anisotropy will decrease. Thus, both rates of rotation and fluorescence emission determine the response in anisotropy (Figure 8A). Methods based on anisotropy are very useful for studying interactions and bindings of smaller molecules bearing a fluorophore to larger molecules since these interactions should result in reduced mobilities of the probes and increased anisotropies. The ratio of the polarized components recorded at two orthogonal polarizations to the total intensity defines anisotropy. As a consequence, it is independent from the absolute fluorescence intensity and fluorophore concentration, giving a direct response to intermolecular interactions. However, one must be careful to exclude the contribution of light scattering that can strongly influence the results. The anisotropy-based technique is also less sensitive than other techniques since the intensity of the excitation beam is reduced due to the use of polarization filters; therefore, higher concentrations of the fluorescent probe are often required to obtain a sufficient signal. Fluorescence anisotropy is widely used for the study of nucleoside–protein and protein–DNA interactions. For the latter, short NA fragments labeled with FNAs are preferred as they exhibit low anisotropy, whereas their binding to a target with larger molecular weight, such as a protein, gives rise to enhanced anisotropy.

To be used in an anisotropy-based assay, a fluorophore must exhibit a robust fluorescence emission with minor sensitivity to the environment. The *N*-Methylanthraniloyl fluorophore (**MANT**) is especially useful in this respect. It is a compact, blue-emitting fluorescent molecule, which can be attached to position 2′ or 3′ of ribose via an ester bond. A series of nucleoside substrates for anisotropy-based studies of nucleotide–protein interactions was developed based on the **MANT** fluorophore. Mocz et al. used 2′- and 3′-**MANT**-labeled ATP (Figure 8B) to study ATP–dynein binding. The fluorescent nucleotides exhibited a modest ≈ 2.2-fold enhancement in fluorescent emission. Meanwhile, the observed anisotropy of the nucleoside–protein complex was as high as 0.38, which is close to the theoretical limit for the absence of rotational mobility. The anisotropy titration data was used to determine the association constants of the complex (Mocz et al., 1998). The binding of **MANT**-modified GTP and GDP with erythrocyte transglutaminase was also monitored by anisotropy (Murthy and Lorand, 2000).

The first experiments employing fluorescence anisotropy for studying of protein–DNA or aptamer–target interactions were performed using DNA molecules labeled with a fluorescent dye, such as fluorescein (Figure 1A), tethered to the 5′ end of the polynucleotide (Ozers et al., 1997; Fang et al., 2001; Li et al., 2007; Zou et al., 2012). The strategy based on 5′-terminal labeling has an intrinsic drawback. Although slower rotational motions of the protein–DNA complex give rise to the measured anisotropy, the fluorophore remains flexible due to the non-rigid nature of the linker attaching it to the sugar-phosphate backbone. The residual local motions of the fluorophore reduce the observed anisotropy and decrease sensitivity of the detection (Hawkins, 2008). Indeed, only about 15% of the linker-attached fluorescein undergoes rotation coupled to global motion of the DNA (Hawkins, 2008). In this respect, a fluorophore with rotational motions that are restricted by incorporation into DNA has a distinct advantage (Shi and Herschlag, 2009).

Accordingly, a FNA must meet several criteria to be suitable for anisotropy-based assays. The fluorophore must be a flat organic molecule in order to minimize its own rotational motions by stacking with natural nucleobases. Furthermore, it must exhibit bright emission insensitive to environmental changes. In an ideal case, the fluorescence quantum yield and lifetime should not change upon binding of the sensor to the target. Bahr et al. investigated the influence of different dye-labeling topologies on the sensitivity of anisotropy-based assays. A DNA-containing the dye tethered to the 5′-end was compared to intercalating dye upon binding to the DNA methyltransferase *M.TaqI*. The ethidium bromide (EtBr) fluorophore was chosen as the base surrogate (Figure 8B; Bahr et al., 2007). The fluorophore was covalently attached to an acyclic deoxyribose moiety and incorporated into DNA via solid-phase phosphoramidite synthesis. Ethidium bromide is known to be an excellent intercalator, exhibiting bright fluorescence when stacked between base pairs in DNA. In addition, its fluorescent emission shows only minor variations in response to the surrounding nucleobase context (Huber et al., 2004). The intensity of fluorescence also remained stable upon binding to the DNA methyltransferases, indicating the absence of non-specific dye–protein interactions. The changes of emission anisotropy were measured upon binding of DNAs to the methyltransferase with the different reporter groups. Using the conjugate-containing fluorophore as a base surrogate gave the best result in terms of increased fluorescence anisotropy. An approximately 2.5-fold increase of anisotropy was observed, which was not observed in non-specific interactions of the protein with fluorophore. Such a strong increase in anisotropy reflects the rotational dynamic of entire protein–DNA complex with minimal contribution of local fluorophore motions (Bahr et al., 2007). These data indicate that an appropriate FNA offers significant advantages over fluorophores attached in other ways.

The bright emissive nucleoside analogs **tC** (Figure 8B) incorporating the nucleobase surrogate 1,3-diaza-2-oxophenothiazine was developed as a cytidine analog (Lin et al., 1995; Wilhelmsson et al., 2001). The tricyclic nucleobase **tC** exhibits interesting properties for anisotropy-based sensing. Base pairing with G, **tC** has a well-defined position and geometry within the DNA double helix, and exhibits bright fluorescence with negligible sensitivity to the surrounding bases (Sandin, 2005). Furthermore, this FNA demonstrates a base-flipping rate that does not interfere with the signal measured in fluorescence anisotropy. The anisotropy of **tC** was used by Wilhemsson et al. to study the incorporation of the analog into DNA by DNA polymerase and to determine in competitive binding essays, the dissociation constant of natural or unnatural nucleotides (Sandin et al., 2009). Pteridine nucleobases can also be employed as fluorescent reporters in anisotropy sensing (Hawkins, 2008). For instance, the **6MI** label (Figure 8B) was effective in anisotropy binding measurements with the *Escherichia coli* protein integration host factor at DNA concentrations of 1 nM and fluorescence intensity measurements at 50 pM DNA (Moreno et al., 2012).

## FNAs for Emission-Shift-Based Sensing

A group of fluorophores is known to red-shift their emission spectra when placed in a medium of increased polarity. This effect is referred to *solvatofluorochromism* and is caused by enhanced dipole–dipole interactions between the excited fluorophore and the surrounding molecules that decrease the excited-state energy (Klymchenko and Mély, 2013). Environment-sensitive fluorophores exhibiting solvatofluorochromic properties are important tools for biophysics and molecular sensing (Klymchenko, 2017). Steady-state and time-resolved fluorescent spectra of these dyes yield information about specific and non-specific interactions with the surrounding molecules, the rates of their dielectric relaxations, etc. Given that they are designed as analogs of amino acids and lipids, such dyes are widely used for probing proteins and lipid membranes (Demchenko et al., 2009; Loving et al., 2010; Krueger and Imperiali, 2013). The general principles governing the performance of these dyes are well-understood. They must contain functional groups attached to aromatic rings that can become strong electron donors and acceptors in the excited state. Being appended to the opposite sides of the π-electronic structure, they generate a strong dipole moment that, by interacting with local molecular environment, results in modulation of the energy of the electronic transition and spectral shift. This shift to longer wavelengths (red shift) is stronger the higher the polarity of the environment. Such a shift can also be modulated by intermolecular H-bonding of one of these groups in a polar environment.

**Figure 9:**
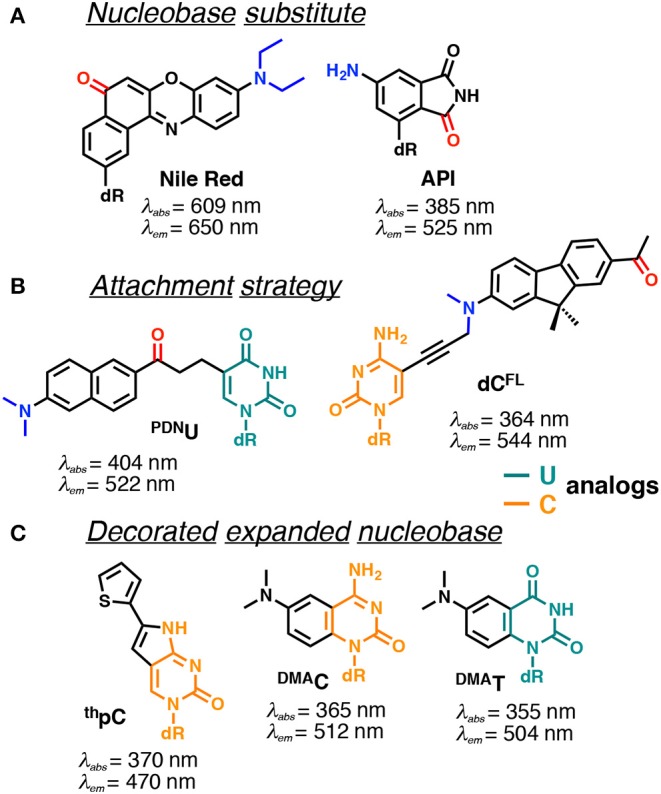
Selected examples of solvatofluorochromic FNAs demonstrating intramolecular charge transfer (ICT): **(A)** employed as nucleobase surrogates; **(B)** covalently and flexibly attached to a pyrimidine; and **(C)** mimicking nucleobases by an appropriate expansion. dR indicates 2'-deoxyribosyl; donor and acceptor groups are depicted in blue and red, respectively (Barawkar and Ganesh, 1995; Okamoto et al., 2006; Tainaka et al., 2007; Noé et al., 2012; Riedl et al., 2012b; Weinberger et al., 2013; Mata and Luedtke, 2015; Mata et al., 2016). Excitation and emission wavelengths were given in nm.

Many efforts have been made to develop FNAs possessing solvent-dependent shifts. The difficulty is the requirement to incorporate the fluorophore into the NA structure, without changing the topology or interactions. Several approaches for the construction of solvatofluorochromic nucleosides have been described (Figure 9). For the first approach, the dye was grafted to deoxyribose, typically via C-glycoside bond, to be further intercalated between nucleobases within the DNA (Okamoto et al., 2006; Weinberger et al., 2013). The second approach requires a fluorescent dye to be linked to the natural nucleobases, typically at position 5 of pyrimidines and positions 7 and 8 of purines (Figure 9B; Kimura et al., 2005, 2006; Tainaka et al., 2007; Riedl et al., 2012b). The third approach features minor conjugated decoration of the expanded nucleobase with additional chemical functionalities endowing with solvatofluorochromic properties (Greco and Tor, 2005; Shin et al., 2011; Noé et al., 2012; Sinkeldam et al., 2012). The latter seems to have a distinct advantage, since minimal perturbations are introduced to the structure of DNA.

**Nile Red** and 4-aminophthalimide (**API**) are among the dyes that exhibit the strongest solvent-dependent shifts of fluorescence spectra (Figure 9A). However, being incorporated into DNA as base substitutes, these dyes showed only a moderate sensitivity to different structural contexts. For instance, upon transition from a single to a double-stranded DNA form, **API** showed only a small 10 nm blue shift of its emission maximum, and the **Nile Red**-based nucleoside did not demonstrate any fluorescence band shift (Weinberger et al., 2013). Only a moderate blue shift was observed upon interaction of DNA labeled with these dyes with β-cyclodextrin (Okamoto et al., 2006). This occurs because the dyes were incorporated in such a way that their π-electronic system interacts mainly with neighboring bases and these interactions do not change substantially upon structural transformations of the DNA.

Probes from the second group contain a fluorophore attached to the nucleobase via a linker, which allows them to be exposed to the major or minor groove of DNA. A series of PRODAN-nucleoside conjugates was developed, where the fluorophore was attached via a short linker to the *N*-4 and *N*-2 exocyclic amino group of cytosine and guanine, respectively (Kimura et al., 2005, 2006) or to the C-5 of pyrimidines (^**PDN**^**U**, Figure 9B) and the C-8 of purines (Tainaka et al., 2007). In contrast to the probes following the first approach, the fluorophore exposed to the major or minor grooves of DNA was more exposed for direct interactions with water and therefore showed significant spectral shifts depending on the NA polymorph in question. Indeed, the label was able to distinguish the polarity of the microenvironment in the minor and major grooves of B-DNA, A-DNA/RNA hybrids and Z-DNA (Kimura et al., 2005, 2006). Okamoto and co-workers extended this approach to the synthesis of the four nucleobases set (U, C, A, G) for single-nucleotide polymorphism genotyping. All the investigated probes showed a substantial increase in fluorescence intensity upon hybridization to a perfectly matched sequence, together with increase in the Stokes shift (Tainaka et al., 2007).

Hocek et al. used a dC analog incorporating a push–pull fluorene fluorophore (**dC**^**FL**^) to sense the protein–DNA interactions as a color change that was distinguishable to the naked eye (Figure 9B). The triphosphate of **dC**^**FL**^ was prepared and incorporated into the DNA using two DNA polymerases. Labeled DNA showed significant changes in the emission wavelength upon interaction with the human transcription factor p53. A substantial blue shift of the emission maximum from 581 to 567 nm was observed upon binding to p53, indicating sufficient screening of the fluorophore from the polar aqueous medium (Dziuba et al., 2016b).

**Figure 10:**
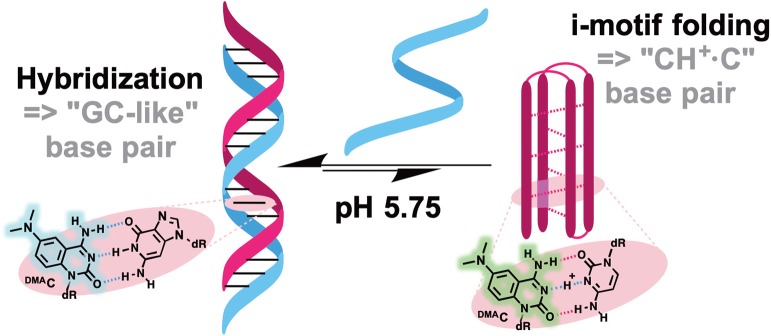
pH-induced i-motif folding monitored by color change of ^**DMA**^**C** fluorescence.

Probes from the third group contain a natural nucleobase core rendered to be solvatofluorochromic by additional chemical functionalities. An example is **5FU**, which was designed for the detection of DNA abasic sites (Figures 1D, 6A). The wavelength of its emission band shifts from 395 nm in diethylether to 431 nm in water (Greco and Tor, 2005). Solvent-dependent emission sensitivity was also observed for a structurally related compound containing thiophene, namely for 6-aza-uridines (Sinkeldam et al., 2012), based on thieno-fused pyrrolo cytosines (^**th**^**pC**, Figure 9C; Noé et al., 2012) and a thieno[3,4-d]-pyrimidine scaffold (Shin et al., 2011). However, being incorporated into polynucleotides, these compounds show only minor shifts in their emission bands, and are more useful for the construction of intensity-based probes. Such behavior is typical for dyes quenched by water, which masks the spectral shift (Demchenko, 2005a). Luedtke et al. designed and synthesized a push–pull FNA composed of dimethylaniline fused to deoxycytidine (^**DMA**^**C**, Figure 9C). ^**DMA**^**C** is a mimic of the natural nucleobase, which, upon pH decrease and base (*N3*) protonation, exhibits remarkable red-shifted absorption and emission maxima. These properties were exploited to study the dynamics of folding and unfolding of i-motif and DNA duplexes of telomeric repeat sequences under real-time conditions (Figure 10; Mata and Luedtke, 2015).

## FNAs for Dual-Band Ratiometric Sensing

**Figure 11:**
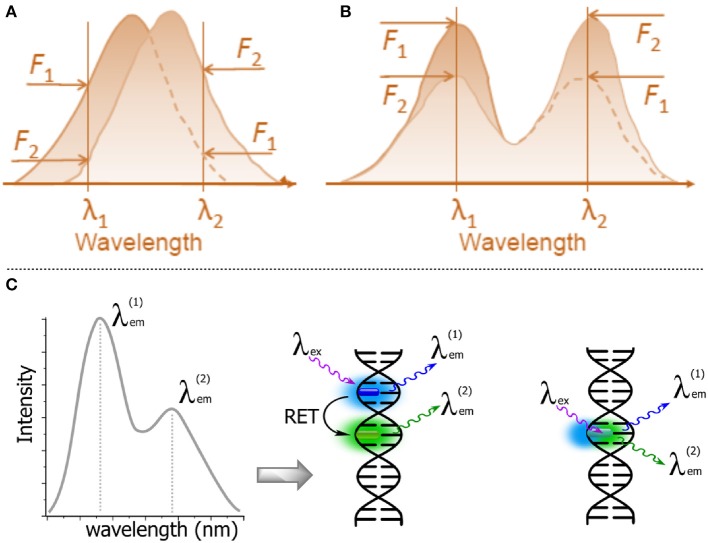
Schematic representations of λ-ratiometric sensing recorded at two fixed wavelengths for a single fluorescent probe. **(A)** The spectral shift defines the sensing response (e.g., Nile Red). **(B)** The decrease of intensity of one band λ_1_ and the concomitant increase of intensity at a different band λ_2_ define the sensing response. Calculating the change in the intensity ratio *F*(λ_1_)/*F*(λ_2_) enables quantitative measurements (Demchenko, 2013). **(C)** Dual emission: two-dyes (e.g., exhibiting resonance energy transfer, RET) vs. a one-dye approach based on the generation of a new band for the recording of a ratiometric signal.

The sensitivity of fluorescence probes to report intermolecular interactions can be greatly increased if the sensing signal is based on the change between two spectrally resolved forms. This is because the wavelength-ratiometric signal will be recorded as a ratio of intensities, not at the slopes of spectral bands but at the band maxima, thus providing a comparison of fluorescence intensities at a higher level and achieving higher wavelength precision (Figure 11A vs. Figure 11B; Demchenko, 2010, 2014). In order to achieve this, the researcher must select a fluorophore that can exist in two ground- or excited-state forms. Moreover, the switching between these forms should occur in the desired range of changes of the intermolecular interactions in question. This approach is fundamentally different from that based on FRET-based switching, which requires the presence of two dyes (donor and acceptor) at a specific distance (Figure 11C; Demchenko, 2014). Such an approach avoids the difficulties arising from double labeling within the same molecular system but it is more challenging, since these properties are very specific and only “smart,” specially designed dyes possess them.

Switching between ground-state forms of such smart fluorophores allows one to extract the effects from fluorescence excitation spectra, and the first λ-ratiometric fluorescent probes for calcium ions were based on this principle (Grynkiewicz et al., 1985). In order to produce interplay between intensities in fluorescence emission spectra, the dye has to be present in two excited-state forms with finely tunable switching between them. An additional requirement is the presence of such two forms of comparable fluorescence intensities. All this makes for a very limited selection of dyes with the appropriate responses.

**Figure 12:**
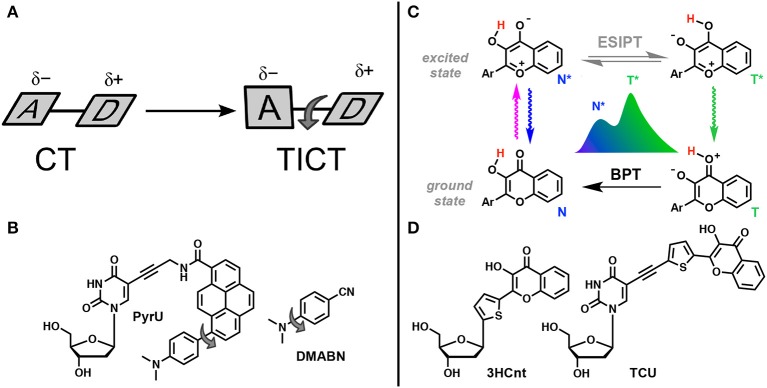
TICT vs. ESIPT. **(A)** Schematic representation of geometrical arrangements of normal charge transfer (CT) and twisted intramolecular charge transfer (TICT) excited states (Rettig, 1986). **(B)** FNA containing fluorophore undergoing TICT reported by Okamoto et al. (2005b). **(C)** Tautomeric equilibrium explaining the dual-band emission of 3-hydroxychromones. ESIPT, excited-state intramolecular proton transfer; BPT, back proton transfer; N* and T* states for normal and tautomer excited forms, respectively. **(D)** Dual-emissive nucleoside analogs based on 3-hydroxychromones (Dziuba et al., 2012; Barthes et al., 2015).

One of the excited-state reactions that can be explored is the intramolecular charge transfer (ICT) that has to be stabilized by isomerization (twisting). Such twisted intramolecular charge transfer (TICT) can drastically modulate the emission spectra depending on the environment, since it is only under conditions of high polarity that the charge-transfer form can be stabilized (Figure 12A). Since the two isomers that appear in the excited state emit light quanta of different energies, they show two well-resolved emission bands. The very large Stokes shift of the long wavelength band due to the emission of the generated excited-state isomer is characteristic for this type of dual probe (Cao et al., 2014; Oesch and Luedtke, 2015).

The other reaction that generates two bands in emission spectra is the excited-state intramolecular proton transfer (ESIPT), which proceeds between proton donor and acceptor groups, closely located in the dye structure (and commonly connected by intramolecular H-bonding, Figure 12C). This reaction is subject to perturbation by the change of polarity in the environment and to the formation of additional intermolecular H-bonds (Han and Zhao, 2010; Tomin et al., 2015).

Fluorophores demonstrating TICT and ESIPT can be introduced as smart NA base analogs that are able to respond to different changes in the properties of their local environment (Figures 12B,D). They have significant potential in the practical sense because the intensity ratio of the two emission bands is highly sensitive to the environment and since single labeling is sufficient for λ-ratiometric detection (*self-calibrated sensing*; Demchenko, 2005b,c).

### Single-Fluorophore Ratiometric Probes Based on TICT

In the TICT system, the donor and the acceptor moieties are joined by a chemical bond (σ or π) and the shift of π-electron density is accompanied by a twist about this bond in the excited state (Figure 12A; Rettig, 1986; Bhattacharyya and Chowdhury, 1993; de Silva et al., 1997; Wu et al., 2011). The prototype and historical example of a TICT fluorophore is 4-dimethylaminobenzonitrile (Lippert, 1955). This planar compound has a well-conjugated push–pull electron system. After light absorption, the initially, locally excited form (LE) has similarly planar geometry and intramolecular relaxation results in twisting, generating the isomer form. In the TICT excited state, the dimethylamino unit becomes orthogonal to the rest of the π-system. Twisting through 90° can achieve full charge separation resulting in an amino cation and a benzonitrile anion and a dipole moment of the TICT rotamer becoming larger that of the LE form. Therefore, an increase in solvent polarity favors the TICT form that, interacting with polar environment, demonstrates a red-shifted emission. Since the TICT form is generated from the rotation between the donor and acceptor segments of the same molecule, restriction to the twisting motion, due to the environment or by increasing the viscosity of the medium, will favor the LE state. On the other hand, temperature increases will favor the TICT state. Whereas, the LE state is emissive, the emission from TICT state is poor in general, which may create a problem for λ-ratiometric analysis.

Okamoto et al. reported the first example of DNA labeled with a dual-emissive TICT probe (Okamoto et al., 2005b). This TICT-based probe was a pyrene derivative electronically coupled to a dimethylamino donor and an amide acceptor (Figure 12B). This probe was tethered to deoxuridine for incorporation into DNA and hybridization with DNA and RNA. While the nucleoside conjugate **PyrU** showed a single-band emission at 540 nm, ss- and ds-ODNs showed dual emission with the appearance of a new band centered at 440 nm. The quantum yield increased together with the dual emission. The fluorescence bands at a shorter wavelength (440 nm) and at a longer wavelength (540 nm) were assigned to the emission of the LE and TICT states, respectively. The TICT emission was largely dominating in ss-ODN, while in ds-DNA, a 1:1 ratio was observed. Therefore, the fluorescent twisted **PyrU** is sensitive to hybridization, as indicated by a change of its color. It was proposed that the equilibrium between the LE and TICT states, and thus the intensity ratio of these two bands, was due to the restricted twisting motion in the ODN environment, which was more pronounced in the duplex. In this case, the steric hindrance and a narrow free space in the duplex acted as a barrier to the internal rotation of the fluorophore instead of a viscous solvent. Recent publications have underlined the effort to find new nucleoside analogs with TICT properties and to extend this new approach for DNA labeling and sensing (Mata and Luedtke, 2013; Suzuki et al., 2014; Schweigert et al., 2017).

### Single-Fluorophore Ratiometric Probes Based on ESIPT

The ESIPT reaction is observed only in those aromatic compounds having proton donor and acceptor groups in close proximity and connected by an intramolecular hydrogen bond. Common proton donors and acceptors are hydroxyl or amino groups and carbonyl oxygen or imino nitrogen, respectively. After photon absorption, the electronic charges are redistributed making the proton donor more acidic and the acceptor more basic, thus favoring the intramolecular proton transfer in the excited state (Zhao J. et al., 2012; Demchenko et al., 2013; Sedgwick et al., 2018).

3-Hydroxychromones (**3HCs**) are typical representatives of ESIPT dyes and are one of the most useful probe families for practical applications. **3HCs** are heterocyclic compounds bearing hydroxyl and carbonyl groups involved in a 5-member H-bonding ring system. Due to ESIPT, **3HC** fluorophores exhibit two excited states, which generate two well-resolved emission bands (Figure 12C; Demchenko, 2006). The short-wavelength band is due to the emission from the normal (N^*^) excited form, whereas the long-wavelength band is produced by the tautomer form (T^*^). In the excited normal state (N^*^), the oxygen carbonyl is more basic, making this group sensitive to the donating ability of hydrogen bond of protic solvents and hydration. As a consequence, increased acidity of the solvent or of the hydration rate favors H-bonding to the carbonyl oxygen resulting in decreased relative intensity of the T^*^ due to a slower ESIPT reaction. Another consequence of the electronic charge distribution for the N^*^ state is related to the dipole moment, which is larger than that for the N^*^ and T^*^ forms. The magnitude of the N^*^ dipole moment can be finely tuned by appropriate chemical modifications, while the dipole moment of the T^*^ state is much less affected. For instance, the electron-rich aryl groups at position 2 (e.g., 4-dimethylaminophenyl) dramatically increase the magnitude of the dipole moment of the N^*^ state due to internal charge transfer (ICT), rendering 3HCs sensitive to polarity change in aprotic solvents. In polar solvents, strong and favorable dipole–dipole interactions between solvent molecules and the dye stabilize the N^*^ state much more than the T^*^ state, resulting in increased emission of the N^*^ state. The N^*^/T^*^ ratio is therefore a robust analytical signal that directly reports on the properties of the microenvironment of **3HC** fluorophore. These features have been used extensively for the construction of environment-sensitive fluorescent dyes to probe proteins and lipid membranes (Klymchenko and Kreder, 2014; Zamotaiev et al., 2014; Sholokh et al., 2015b).

**3HCs** bearing a thienyl or furyl ring at position 2 are known to be extremely sensitive to hydration. The emission switches from the dominating N^*^ band in water to T^*^ emission in aprotic media. **2-Thienyl-3HC** was therefore selected for DNA labeling. It was formulated as a deoxyribose derivative (**3HCnt**, Figure 12D) and incorporated into DNA via the standard phosphoramidite method. The dye showed a dominant tautomer (T^*^) emission in the single-stranded form. A further increase of the T^*^ band emission was typically observed upon hybridization to a complementary strand (Dziuba et al., 2012). Minimal DNA perturbation by the dye incorporation and its intercalation mode were supported by CD, Tm and NMR investigations (Zargarian et al., 2017). Importantly, the emission of single-stranded DNA was highly sensitive to the formation of the complex with the HIV-1 nucleocapsid protein (NC). A strong increase of the N^*^ emission was observed, indicating that the **3HC** nucleoside can be a good sensor for protein–DNA interactions (Dziuba et al., 2012). The **3HC** label was employed to study the conversion of the (–)DNA copy of the HIV-1 primer binding site (–)PBS stem-loop into the (+/–)PBS duplex in the absence and presence of the NC chaperone protein. In contrast to **2AP**, the **3HC** probe provided the first complete mechanistic description of this critical process in HIV-1 replication (Sholokh et al., 2018). In a comparison study with commercially available FNAs, the fluorescent label was exploited to investigate the mechanism of the DNA repair enzyme, endonuclease VIII, in interactions with damaged DNA. The results of this study showed that this label exhibits higher sensitivity and yields more information about the conformational changes of DNA binding and processing. Using **3HC**-based molecular probes, the kinetic mechanism of action of endonuclease VIII was specified (Kuznetsova et al., 2014). The FNA was also successfully employed to record the stepwise binding of the ubiquitin-like domains 1 containing PHD and RING finger (UHRF1) followed by the flipping of a 5-methylcytosine (5 mC, Figure 5B). The increased lifetime of UHRF1 bound to DNA containing 5 mC supports the idea that UHRF1 is the key partner for the recruitment of DNA methyltransferase 1 to the correct site for faithful replication of the DNA methylation pattern (Kilin et al., 2017).

The internal **3HC** base surrogate proved to be extremely sensitive to proximal disturbances, although its sensitivity to hydration changes was not optimal. Probes sensitive to hydration are desirable for sensor development because the interactions between proteins and other ligands, as well as structural changes, affect the proximal distribution of water molecules around the NA (Barthes et al., 2016). The possibility to connect the 3HC dye to uracil and adenine nucleobases for external labeling of NA (for example, in the major grooves of DNA duplexes) has been also explored (Dziuba et al., 2014; Barthes et al., 2016; Le et al., 2019). The new conjugated nucleobase–**3HC** fluorophores demonstrated strong sensitivity to hydration (Barthes et al., 2015; Le et al., 2019). DNA strands incorporating the emissive deoxyuridine analog were synthesized and studied (Barthes et al., 2016; Gavvala et al., 2016), and the resulting probe exhibited two-color emission and provided high sensitivity allowing discrimination between matched and mismatched DNA duplexes, and B-DNA/DNA and A-DNA/RNA forms (Barthes et al., 2016). The change between these different forms was easily identified by a color change due to the variation of N^*^/T^*^, which was attributed to the change of hydration in the proximity of the fluorescent base reporting group. Compared to the wavelength shift of the dimethylaminonaphtalene (dan) fluorophore, which displayed only a 5% variation in the λ-shift of the emission maximum (Kimura et al., 2005), the dual-emissive uracil probe demonstrated about 11-fold enhanced sensitivity to detect the B to A transition with a 55% variation of its N^*^/T^*^ ratio.

## Conclusions and Perspectives

FNAs have become indispensable tools in biophysics and analytical chemistry. Despite significant progress and the large number of FNAs reported to date, new efforts continue and further developments in this field are expected (Manna et al., 2018; Sabale et al., 2018). As the reader may have noticed, the intensity-based probes are over-represented in the literature, whereas less attention has been paid to FNAs that exploit other reporting principles. We expect further progress in the development of FNAs that use the prospective reporting modes, described above.

Rapid progress is expected in the application of molecular probes based on FNAs in complex biological media, such as living cells. Given their minimal perturbation of DNA or RNA structures, fluorescent hybridization probes can enable visualization of these molecules, providing information on their location and transportation, but they can also reveal dynamics and interactions with cellular components. Ideally, fluorescence response should be increased to a level that does not require any target amplification, purification or removal of unreacted probes. Strong similarity to natural nucleosides and the minimal perturbance from their interactions with complementary bases allow for high specificity. Moreover, the possibility to assess polarity- and H-bond-sensitive responses in these smartly designed probes yields deeper understanding of NA structure, dynamics and interactions at the sub-molecular level. Because of their native-like linkage to DNA or RNA, these probes are very closely associated with neighboring bases, rendering them exquisitely sensitive to the subtle changes that occur in the atomic-level structure surrounding them. Therefore, changes in base stacking or base pairing in the vicinity of these probes are reflected by distinct changes in fluorescence response. Thus, new concepts and probes demonstrating advanced properties have a promising future.

The majority of known nucleoside analogs are excited by UV and blue light, and are therefore prone to photodamage and interference from autofluorescence, which might have to be overcome in future molecular design strategies. For this reason, new nucleosides that are excitable at longer wavelengths are highly desirable. Alternatively, the situation can be improved by the development of two-photon excitable FNAs. Two-photon absorption (2PA) is a physical process where the chromophore is excited by the simultaneous absorption of two low-energy photons, which match the energy of a single photon of higher energy that corresponds to normal excitation. Fluorophores with high 2PA cross-sections are of great importance for fluorescence microscopy and intracellular studies, because the two-photon excitation decreases the background signal caused by autofluorescence and reduces the sample photodamage. The existing FNAs possess quite low 2PA cross-sections. For instance, the measured values of **2AP**, pteridines and **tC** are in the range between 0.2 and 3.5 GM units (Stanley et al., 2005; Lane and Magennis, 2012). Consequently, FNAs with higher 2PA are expected to appear in the future, which will open new possibilities in two-photon microscopy. Likewise, this will enable single-molecule detection to become more efficient within the living cells.

FNAs will not only be used as passive fluorescence emitters, but will also perform additional functions. Several literature reports have shown that fluorescently labeled NAs can be used as a dual probe for fluorescence detection combined with electron microscopy (Holzhauser et al., 2013) or NMR spectroscopy (Sabale et al., 2018). In the first study, the fluorophore used induced photochemical polymerization of 3,3′-diaminobenzidine, enabling electron microscopy detection. In the second example, the dual probe combines a fluorescent molecular rotor and a ^19^F label allowing detection and resolution of the topologies of G quadruplexes in cells. Another attractive possibility is the use of FNAs as light-controlled molecular triggers. Site-selective incorporation of photochemically reactive nucleoside makes it possible to change the conformation and chemical structure of NAs by light; for example, light-controlled hybridization (Ogasawara and Maeda, 2008) or controlled formation of inter-strand cross-links (Haque et al., 2014). Finally, the integration of FNAs into functional NAs could be a powerful strategy for the creation of light-guided catalytic systems. An example is the **6MI**-modified DNAzyme that can harness violet light to repair DNA damages (Barlev and Sen, 2013). New examples of FNAs that are used in the rational design of light-controlled molecular systems are expected to appear in the future.

## Author Contributions

DD and BM produced figures. DD, BM, and AB wrote the sections Operation With Parameters of Fluorescence Emission, FNAs for Intensity-Based Sensing, FNAs for Lifetime-Based Sensing, FNAs for Anisotropy-Based Sensing, FNAs for Emission-Shift-Based Sensing, and FNAs for Dual-Band Ratiometric Sensing. RB wrote the section Incorporation of Fluorescent Nucleoside Analogs (FNAs): Methodology. AD, DD, BM, and AB wrote the sections Introduction, Resolving Problems With Smart Base Substituents, Incorporation of Fluorescent Nucleoside Analogs (FNAs): Methodology, and Conclusions and Perspectives.

### Conflict of Interest

The authors declare that the research was conducted in the absence of any commercial or financial relationships that could be construed as a potential conflict of interest.
